# Refractory serositis in Gorham–Stout syndrome

**DOI:** 10.1186/s13023-022-02307-8

**Published:** 2022-04-04

**Authors:** Hong Di, Bingqing Zhang, Na Xu, Yue Yin, Xinxin Han, Yun Zhang, Xuejun Zeng

**Affiliations:** Department of Family Medicine & Division of General Internal Medicine, Department of Medicine, Peking Union Medical College Hospital, Chinese Academy of Medical Sciences, State Key Laboratory of Complex Severe and Rare Diseases (Peking Union Medical College Hospital), Beijing, 100730 People’s Republic of China

**Keywords:** Bisphosphonates, Clinical characteristics, Gorham–Stout syndrome, Lymphatic malformations, Prognosis, Serous effusion, Sirolimus

## Abstract

**Background:**

Gorham–Stout syndrome (GSS) is a rare disorder with various presentations and unpredictable prognoses. Previous understandings of GSS mainly focused on progressive bone destruction, while we identified a group of GSS patients with serous effusion as the first symptom. This study aimed to investigate the clinical characteristics of patients with GSS having serous effusion as the first symptom.

**Methods:**

Patients diagnosed with GSS were identified through the Peking Union Medical College Hospital Medical Record System. The demographic, clinical, laboratory, and imaging data were collected. Patients who first presented with serous effusion were recruited into the serous group, while those with bone destruction were recruited into the bone group.

**Results:**

Of the 23 patients with GSS enrolled, 13 were in the bone group and 10 in the serous group. The median disease duration was shorter and exercise tolerance was lower in the serous group. Despite less frequent bone pain in the serous group, the frequency of bone involvement was similar to that in the bone group. Patients in the serous group had higher rates of bilateral pleural effusion and multiple serous effusion. However, serous effusion also developed with disease progression in the bone group. Of the 17 patients treated with bisphosphonates, 14 reached bone-stable state. However, 5 out of 10 patients with serous effusion still had refractory effusions after bisphosphonates treatment. Three patients received sirolimus treatment, with an improvement in serous effusion. Seventeen patients were followed up; three patients died, two in the bone group and one in the serous group.

**Conclusions:**

This study discovered that GSS could first be presented with serous effusion. We believe that this may be a new phenotype of the disease. Sirolimus might help in controlling serous effusion and improving prognosis.

## Introduction

Gorham–Stout syndrome (GSS), is a rare and debilitating disease characterized by benign vascular–lymphatic hyperplasia and progressive osteolysis. It is also known as massive osteolysis, vanishing bone disease, or phantom bone disease. It occurs mostly in children and middle-aged people, and may affect multiple systems. This disease was first reported in 1831 [1]. Then in 1955, Gorham and Stout summarized the disease for the first time [2], describing the association between vascular–lymphatic hyperplasia and osteolysis. The etiology and pathogenesis of GSS are not well understood yet. Several hypotheses have been proposed, for example, vascular endothelial growth factor (VEGF), interleukin-6 (IL-6), osteoclasts, and so forth are likely related to the onset of GSS [3–10].

Only about 300 scattered cases were reported worldwide, and the clinical scenario varied significantly. Apart from vascular–lymphatic hyperplasia, bone erosion was another main symptom, leading to pain and pathological fractures. Other reported symptoms included serous effusion, dyspnea, anemia, and abnormal coagulation. The diagnosis of the disease was difficult and might require a combination of clinical, radiological, and pathological features after excluding other diseases [11]. No standard treatment was available for GSS, and the prognosis varied across different reports. Some believed that the prognosis depended on the systems involved [12–14]. For example, patients with pleural effusion might have a poorer prognosis.

We found in recent years that some patients had refractory serous effusion as the main manifestation of GSS, and the incidence was significantly higher than that in previous reports. Even some patients had serous effusion as the first symptom, and the manifestation of bone destruction was more insidious. Therefore, in this study, we summarized the clinical manifestations, radiological characteristics, treatment effects, and prognoses of the largest case series of GSS in the existing reports to provide further knowledge of the disease and clues for future exploration.

## Materials and methods

This study was approved by the Institutional Review Board of Peking Union Medical College Hospital (PUMCH) and conducted in compliance with the Declaration of Helsinki.

The PUMCH electric medical record system was searched using the following keywords: “Gorham–Stout syndrome,” “Gorham Stout disease,” “Gorham syndrome,” “massive osteolysis,” “vanishing bone disease,” and “lymphangioma disease”; the time range was January 01, 1983, to December 31, 2021.

The clinical data, including patient demographics, clinical presentations, laboratory results, and pathological and radiological examinations, were collected. Bone destruction was evaluated using serum C-terminal cross-linking telopeptide of type I collagen (β-CTX) and total 25-hydroxyvitamin D (T25-OHD), bone mineral density, whole-body bone scan, and bone biopsy. Serous effusion was evaluated by chest and abdomen imaging and lymphatic imaging. The clinical data were assessed by two independent investigators after training. Different opinions were consulted with a senior supervisor.

The inclusion criteria by Heffez [11] were as follows: (1) positive biopsy findings in terms of the presence of angiomatous tissue; (2) absence of cellular atypia; (3) minimal or no osteoclastic response and absence of dystrophic calcifications; (4) evidence of local progressive bone resorption; (5) nonexpansive, nonulcerative lesions; (6) absence of visceral involvement; (7) osteolytic radiographic pattern; and (8) negative hereditary, metabolic, neoplastic, immunologic, and infectious etiology. Also, cases with unclear diagnosis were excluded.

The patients were divided into two groups according to the initial onset symptoms. Patients with the first symptom of bone destruction were recruited into the bone group, while patients with the first symptom of serous effusion were recruited into the serous group.

The disease duration was defined as the time from the onset to diagnosis; leukopenia was defined as the number of white blood cells less than 4.0 × 10^9^/L; anemia was defined as the hemoglobin level lower than 120 g/L in men or 110 g/L in women; thrombocytopenia was defined as the number of platelets lower than 100 × 10^9^/L; hypocalcemia was defined as the calcium level lower than 2.1 mmol/L; hyperphosphatemia was defined as the phosphate level higher than 1.4 mmol/L; the normal range of β-CTX was between 0.21 and 0.44 ng/mL; and the normal range of T25-OHD was between 8.0 and 50.0 ng/mL. The bone lesion was defined as the loss of cortical bone in the radiological examinations.

Follow-ups were carried out in outpatient clinics or by telephone, including the change in clinical manifestations, laboratory examinations, radiological examinations, and treatment after discharge. Stable was defined as no expansion in bone lesions and serous effusion. Progression was defined as the worse condition of bone lesions and serous effusion.

### Statistical analyses

Statistical analyses were performed using IBM SPSS Statistics version 24.0 software (IBM, NY, USA). Continuous variables were reported as the mean ± standard deviation for normally distributed data or the median (interquartile range) for non-normally distributed data. Normally distributed continuous variables between two groups were analyzed using independent-samples *t* tests, the median values between the two groups were compared using the Kruskal–Wallis test, and categorical data were analyzed using the chi-square test or Fisher’s exact tests. A two-tailed *P* value < 0.05 indicated a statistically significant difference.

## Results

A total of 56 patients were found during the medical record search. Further, 33 patients were excluded due to acroosteolysis, generalized lymphatic anomaly, or unclear diagnosis. Finally, only 23 patients were enrolled into the study. Based on their primary presentation, 13 patients were in the bone group and 10 in the serous group. The detailed demographic and clinical data are shown in Table 1.

**Table 1:** Demographic features, clinical features, laboratory parameters, and treatment of 23 patients with GSS

	Total (*n* = 23)	Bone onset group (*n* = 13)	Serositis onset group (*n* = 10)	*P* value
Age of disease onset (mean ± SD) (year)	24 ± 11.7	24 ± 14.3	24 ± 7.7	0.855
Male/female ratio	1:1.56	1:2.25	1:1	0.417
Disease duration, [median (range)] (year)		4.5 (1.29–22)	1.335 (1–8)	0.131
History of trauma (*N*, %)	5 (22%)	5 (38.5%)	1 (10.0%)	0.179
Clinical features (*N*, %)				
Bone pain	14 (61%)	12 (92.3%)	2 (20.0%)	0.001*
Skeletal deformity	3 (13%)	3 (23%)	0	0.229
Pathological fracture	3 (13%)	3 (23%)	0	0.229
Soft tissue swelling	4 (17%)	1 (7.8%)	3 (30%)	0.281
Decreased exercise tolerance	9 (39%)	2 (15.4%)	7 (70%)	0.013*
Dyspnea	3 (13%)	1 (7.8%)	2 (20%)	0.56
Involvement of skeletal system [median (range)]				
No. of bone involvement	2 (1–3)	2 (1–3.5)	2 (1–3)	0.771
Involvement of vascular system (*N*, %)				
No. of vascular malformations	9 (39%)	1 (7.7%)	8 (0.8)	0.01*
Serous effusion	13 (56%)	3(23%)	10 (100%)	0.00*
Pleural effusion	13 (56%)	3 (23%)	10 (100%)	0.00*
Ascites	3 (13%)	0	3 (30%)	0.068
Pelvic effusion	4 (17%)	0	4 (40%)	0.024*
Pericardial effusion	2 (8.7%)	0	2 (20%)	0.178
Laboratory parameters (*N*, %)				
Leukopenia	1/23 (4.3%)	0/13	1/10 (10%)	0.435
Anemia	2/23 (8.7%)	0/13	2/10 (20%)	0.178
Thrombocytopenia	2/23 (8.7%)	0/13	2/10 (20%)	0.178
Coagulative dysfunction^$^	6/18 (33%)	1/9 (11%)	5/9 (56%)	0.131
Hypocalcemia	7/23 (30.4%)	5/13 (38%)	2/10 (20%)	0.405
Hyperphosphatemia^$^	6/19 (31.5%)	5/11 (45%)	1/8 (12.5%)	0.17
Elevated β-CTX^$^	7/13 (54%)	1/7 (14%)	6/6 (100%)	0.005*
Reduced T25-OHD^$^	4/13 (31%)	0/8	4/5 (80%)	0.007*
Treatment (*N*, %)				
Bisphosphonates	17 (74%)	10 (78%)	7 (70%)	1.00
Sirolimus	3 (13%)	0	3 (30%)	0.068
Surgery	9 (39%)	3 (23%)	6 (60%)	0.086
Radiotherapy	2(15%)	2 (15%)	0	0.308

*Represents statistical significance

^$^Represents missing data in the corresponding variables

Among the 23 patients with GSS, 9 were male (39.1%), and 14 were female (60.9%); no sex-related difference was found in our patients. The average age at diagnosis and the disease duration were consistent with those in previous studies. The disease duration was relatively shorter in the serous group [1.3 (1–8) vs. 4.5 (1.29–22)] compared with the bone group, suggesting that the disease progression was more rapid in this group. The past and family histories were unremarkable in all patients.

In terms of general presentation, seven patients (70%) in the serositis group had decreased exercise tolerance, which was significantly higher than that in the bone destruction group (15.4%, *P* value 0.013). Besides, two patients in the serous group had dyspnea and two had anemia; the incidence of both of them was also higher than that in the bone group.

### Clinical symptoms of GSS

Multi-modal imaging studies of the 23 patients showed that 12 had image-evidenced vascular–lymphatic malformation, 2 in the bone group and 10 in the serous group. Among the two patients in the bone group, one had thoracic duct obstruction. Among the 10 patients in the serous group, 3 had thoracic duct rupture, 4 had thoracic duct obstruction, and 1 had diffused lymphatic dilatation of the right pleura with local compression and stenosis of the thoracic duct.

**Fig. 1:**
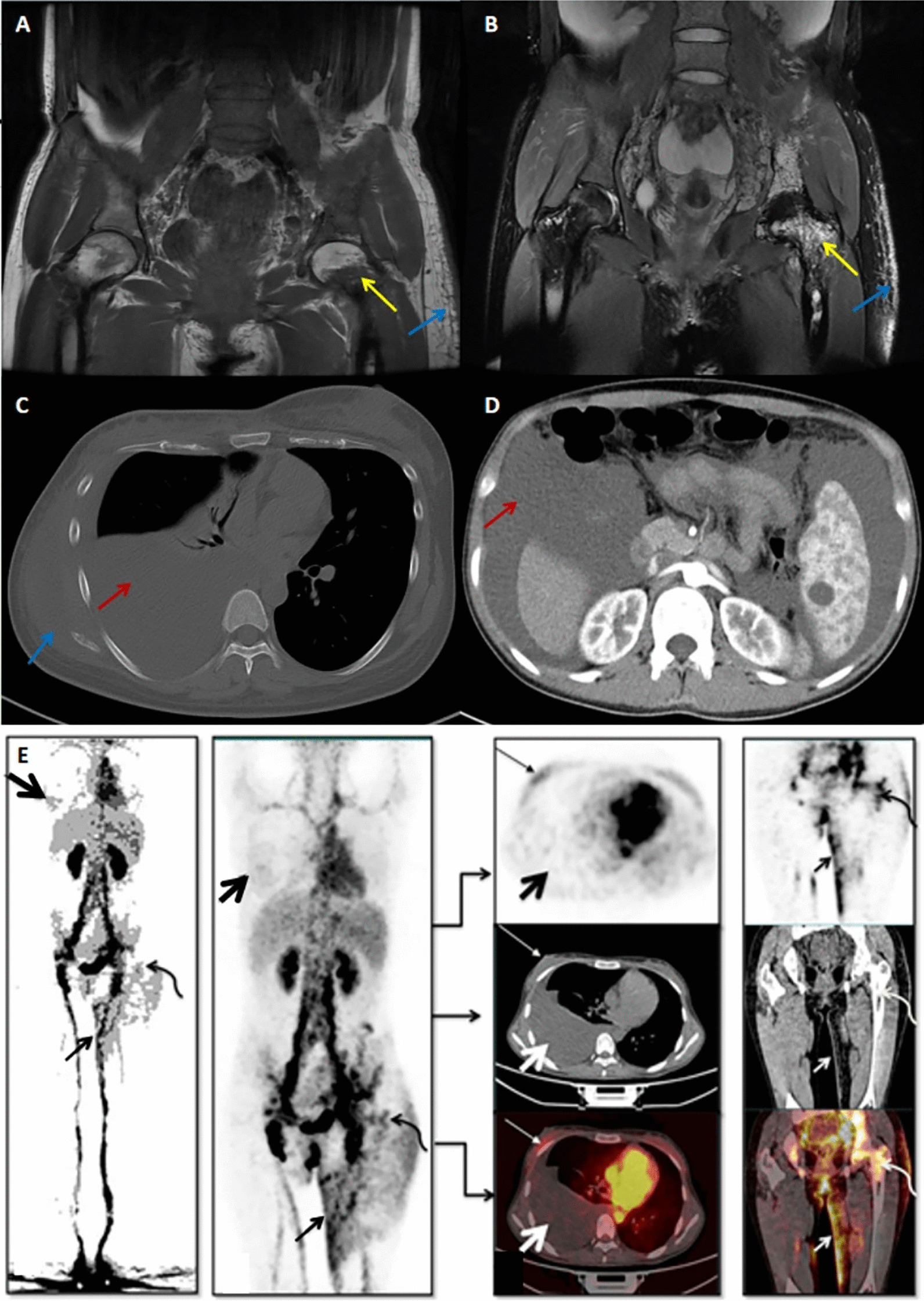
Characteristic imaging findings of GSS. **A** and **B** Bone lesion (yellow arrows) of the left femur and the incrassated subcutaneous tissue (blue arrows) of the left hip on MRI T1 and T2 images. **C** and **D** Massive pleural and peritoneal effusion, respectively (red arrows), and the incrassated subcutaneous tissue (blue arrows) of the right chest wall on CT images. **E** Increased uptake of ^68^Ga-NEB in femur (thin, curved arrow), pleural effusion (thick, straight arrow) and skin (thin, straight arrow) on ^68^Ga-NEB-PET/CT. MRI, Magnetic resonance imaging; CT, computed tomography; ^68^Ga-NEB-PET/CT, ^68^Ga-NOTA-Evans Blue-positron emission tomography/computed tomography

**Fig. 2:**
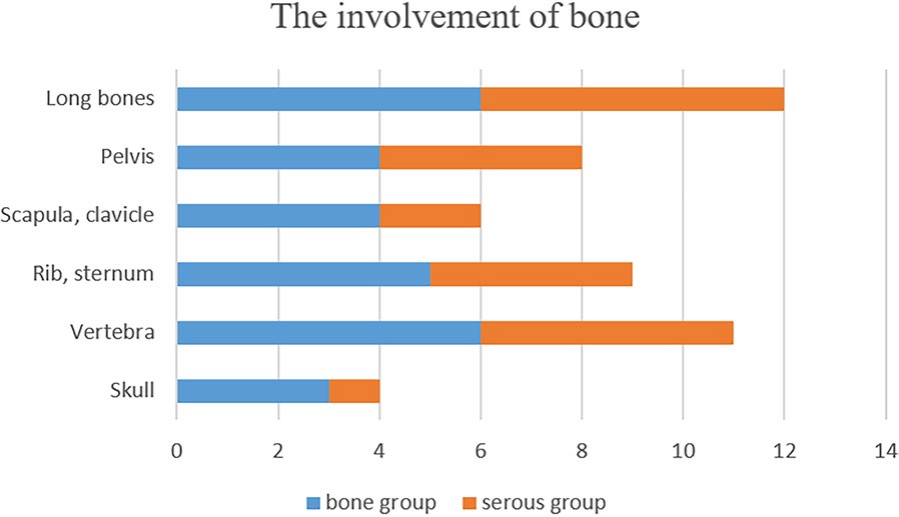
Bone involvement in different parts (N = number of patients who had the specific bones involved). Bone group represents the patients first presenting with bone destruction; serous group represents the patients first presenting with serous effusion

Bone lesion is the most common manifestation of GSS. Figure 1 a and b shows the typical osteolysis in GSS. The long bones were the most common site of involvement. The specific bone involvement in the 2 groups is shown in Fig. 2. A total of 14 patients (61%) had bone pain, which was significantly higher in the bone group (92.3%) than in the serous group (20%, *P* value 0.001). The average frequency of bone involvement evidenced by imaging studies was 2, which was similar in the two groups. With regard to the levels of serum markers, 100% (6/6) of patients in the serositis group had an increased β- CTX level and 80% (4/5) of patients had a decreased T25-OHD level, which were significantly higher than those in the bone destruction group (100% vs. 14% and 0% vs. 80%, respectively, *P* value 0.007 and 0.005, respectively). On the contrary, three patients (13%) had skeletal deformity and another three patients (13%) had pathological fractures in the bone group.

Serous effusion is the second common manifestation of GSS. Thirteen patients (56%) in our study showed serous effusion, 10 in the serous group (100%) and the other 3 in the bone group (23%). For three patients in the bone group, the time from the occurrence of bone lesions to the occurrence of serous effusion was 8 years, 15 years, and 7 years, respectively; two of them showed unilateral pleural effusion, and the other one showed bilateral pleural effusion. Among the 10 patients in the serous group, 4 (40%) had bilateral pleural effusion and 5 (50%) had serous effusion at 2 sites or more. Regarding the properties of serous effusion, eight patients (62%) had bloody and chylous effusion, four (31%) had pure chylous effusion, and one (7%) had pure bloody effusion. Figure 1 c and d shows the obvious serous effusion in GSS.

In terms of the other manifestations for GSS, two patients in the serous group had localized intravascular coagulopathy (LIC), manifested as anemia, thrombocytopenia, and coagulation dysfunction, and also had recurrent and refractory serous effusion.

Apart from lymphoscintigraphy, ^68^Ga-NOTA-Evans Blue-positron emission tomography/computed tomography (^68^Ga-NEB-PET/CT) was conducted in three patients in the serous group who had refractory serous effusion. It could simultaneously and more comprehensively show the location of lymphatic abnormalities in bone, serous cavity, liver, spleen, and so forth. Figure 1e shows the increased uptake of ^68^Ga-NEB in femur, pleural effusion, and skin of one patient.

### Treatment and prognosis

Of the 17 patients who received traditional treatment of bisphosphonates (BPs) and calcium agents, 10 (58.8%) were in the bone group, and 7 (41.2%) in the serous group. Bone pain improved in all 17 patients after treatment. One patient (5%) in the serous group had partial recovery of previous bone destruction evidenced by bone imaging, 14 (70%) were in stable condition, and 2 (10%) had worsened bone destruction during follow-up.

However, after receiving BP treatment, 50% (5/10) of patients with GSS still had recurring serous effusion, four in the serous group and the other one in the bone group. Sirolimus was used in three of the five patients in addition to BPs. All three patients had image-evidenced effusion recession, and the general conditions of the three patients significantly improved.

In total, 17 patients were followed up. Of these, 11 (64%) were in a stable condition, 3 (18%) had progression, and another 3 (18%) patients died. One patient in the serous group died of refractory pleural effusion and dyspnea even after pleural adhesion therapy. One patient in the bone group died of progressive bone destruction even after BP treatment. The other patient in the bone group died of unknown causes.

## Discussion

GSS is one of the lymphatic malformations (LMs) [15]. Compared with other LMs, GSS is characterized by progressive bone destruction; some patients also show serous effusion. This study was the largest single-center case series of GSS worldwide reporting the cases of 23 patients with GSS. It was performed on a group of patients with GSS with serous effusion as the first symptom.

The onset of age, sex ratio, and family history of our cohort were all consistent with previous reports. However, the disease duration was relatively shorter in the serous group than in the bone group. Despite no statistically significant differences between the two groups, the patients in the serous group had more rapid disease progression.

Bone pain is the most common manifestation of GSS. Bone pain was significantly less frequent in the serous group than in the bone group (20% vs. 92.3%, *P* value 0.001). However, the average number of bone involvement (median = 2) was similar in the two groups. The levels of serological markers of bone destruction (β-CTX and T25-OHD) were higher in the serous group, suggesting a more insidious bone destruction process in this group. Therefore, it was prudent to evaluate bone involvement in patients with GSS even without bone pain.

Previous studies reported that about 17–25% of patients with GSS also had pleural effusion [16, 17]. However, in our study, 54% (13/23) of patients with GSS showed serous effusion, 10 in the serous group and the other 3 in the bone group. Compared with patients in the bone group, more patients in the serous group tended to have bilateral effusion and more than one effusion location; also, patients in the serous group tended to have lower activity tolerance. Thus, patients with serous effusion would have worse quality of life. On the contrary, three patients in the bone group also had serous effusion during their disease progression. Therefore, clinicians should evaluate serous effusion during the disease follow-up. Further, we should consider whether patients with unexplained bloody and(or) chylous serous effusion have GSS or other diseases of LMs for the early detection and diagnosis of disease.

The mechanisms of serous effusion are still unknown. Some suggested the association of VEGF, a vital factor in lymphangiogenic process [18–20], with the pathogenesis of GSS [3–6]. Some others reported an elevated plasma VEGF level, which decreased after treatment [10, 16, 17, 21–28]. In addition, increased plasma IL-6 levels might also contribute to the pathogenesis of GSS [7, 8, 10]. In our study, one patient in the serous group had a normal VEGF level, and two of three patients (66.7%) had elevated IL-6 levels. Our study found that patients in the serous group had more and severer lymphatic malformation compared with patients in the bone group. Thus, the serous effusion might be attributed to the severer lymphatic malformation.

Among 23 patients with GSS, 2 adult female patients had anemia, thrombocytopenia, and coagulation dysfunction (including low fibrinogen level, increased D-dimer level, and fibrinogen degradation products), leading to LIC. These two patients were in the serous group, and had voluminous and recurring pleural effusion and ascites. Their diseases progressed rapidly and were difficult to control. Anemia might be due to the lymph leakage (chylothorax and ascites) [29]. LIC was speculated to be associated with abnormal platelet activation by abnormal vascular or lymphatic endothelium entrapping. It was characterized by the elevation of the D-dimer level and fibrin degradation products, low levels of fibrinogen, and sometimes mild-to-moderate thrombocytopenia [30]. It could increase the risk of intralesional thrombosis and severe peri-surgical/procedural hemorrhage [31]. It was commonly reported in venous malformation, lymphatic-venous malformations, or other slow-flow vascular malformations, as well as in complex lymphatic anomalies (CLA) [29], but not in GSS earlier. These two cases suggested that coagulation should be carefully evaluated in the case of LIC and its complications for patients with GSS having refractory serous effusion. More attention should be paid to the coagulation-related indicators; once LIC occurs, one should be alert to thrombosis and severe hemorrhage.

Regarding the novel examination of ^68^Ga-NEB-PET/CT, in previous studies [32, 33], ^68^Ga-NEB-PET/CT revealed the structure of lymphatic vessels more clearly and comprehensively compared with ^99 m^T-ASC lymphoscintigraphy, and the imaging time was significantly shorter. Its 3D imaging mode was more sensitive to the discovery of abnormalities in the lymphatic system, which helped improve the sensitivity and accuracy of the examination. Therefore, it might play an important role in diagnosing and evaluating GSS.

No standardized treatment strategy is available due to the rarity of GSS. The traditional medicine therapy includes BPs, calcium, vitamin D, and so forth [8]. BPs could inhibit osteolysis by restraining osteoclast-mediated bone resorption. Previous studies showed that BPs or BPs combined with vitamin D could improve bone destruction in patients with GSS [34, 35]. In our study, the bone destruction progression was improved or steady in 88% of patients (15/17) after the treatment of BPs, and the effective rate was 100% (7/7) and 80% (8/10) in the serous and bone groups, respectively. Thus, BPs were effective against bone lesions in both groups. Severe bone destruction could be treated with surgical dissection, bone grafting (bone transplantation), or radiotherapy [36]. Six patients in our study had bone-related surgery combined with the treatment of BPs, and their bone structures remained stable after the surgery.

Despite the excellent efficacy of BPs in bone destruction, their effect on serous effusion is not satisfactory. Five out of 10 (50%) patients in our study showed repeated serous effusion after BP treatment. Pleurodesis, ligation of thoracic duct were reported with various effects [14, 36]. In recent years, given the finding that somatic/germ cell mutations of RAS/MAPK/ERK and PI3K/protein kinase B/mTOR pathways might be involved in vascular malformation, mTOR inhibitor sirolimus was used in patients with LMs [37, 38]. In a prospective study on LMs, Adams et al. reported an excellent therapeutic effect of sirolimus in controlling vascular malformation and improving patients’ quality of life [39]. In our case series, three (30%) patients in the serous group received sirolimus 1–2 mg once per day along with BPs. All three patients had controlled or reduced serous effusion; this incidence was significantly higher than the incidence in those without sirolimus combination therapy. Therefore, it was suggested that sirolimus might be effective against serous effusion in GSS.

The mortality of GSS reported earlier varied from 13 to 43.6% [34], depending on the severity of the disease. In our study, 3 of 17 patients who were followed up died, 2 (2/10, 20%) in the bone erosion group and 1 (1/7, 14%) in the serous group. One patient in the bone erosion group died of unknown causes. The other patient in the bone group developed serous effusion during follow-up and died of progression. The one patient in the serous group died of dyspnea.

Our study also had certain limitations. First, it had inevitable systemic bias due to its single-center retrospective nature with limited sample size. However, clinical instincts might still be valuable for clinicians due to the rarity of the disease. Second, patients still required longer follow-up to fully understand the disease progression. Third, this was a descriptive clinical study. Mechanical investigations of the disease, particularly the causes of refractory serous effusion, would be performed in the future.

## Conclusions

In conclusion, we reported 23 patients with GSS to raise awareness of this rare disease. We also discovered that the disease could first be presented with serous effusion. Patients first presenting with serous effusion had shorter disease duration and more insidious bone destruction, we believed this may be a new phenotype of the disease. In addition, serous effusion might occur during disease progression and contribute to worse prognosis. Although BPs were effective in controlling bone destruction, the effect of BPs on serous effusion was not satisfactory. Other therapies, such as sirolimus, might still be beneficial against refractory serous effusion. Patients with GSS should be evaluated thoroughly and be followed up continuously for the early detection of the disease fluctuation. Further bench and bedside investigations of the disease would be required for better understanding of the disease and for improving the clinical management of patients.

## Data Availability

The corresponding author will, on request, detail the restrictions and any conditions under which access to some data may be provided.

## Abbreviations

GSS: Gorham–Stout syndrome
CTX: C-terminal cross-linking telopeptide of type I collagen
T25-OHD: Total 25- hydroxyvitamin D
BPs: Bisphosphonates
Hb: Hemoglobin
Plt: Platelet
IL-6: Interleukin-6
ESR: Erythrocyte sedimentation rate
CRP: C-reactive protein
LIC: Localized intravascular coagulopathy
LMs: Lymphatic malformations
VEGF: Vascular endothelial growth factor
PTH: Parathyroid hormone
mTOR: Mammalian target of rapamycin
MRI: Magnetic resonance imaging
CT: Computed tomography
^68^Ga-NEB-PET/CT: ^68^Ga-NOTA-Evans Blue-positron emission tomography/computed tomography
